# Preschool Children’s Loose Parts Play and the Relationship to Cognitive Development: A Review of the Literature

**DOI:** 10.3390/jintelligence11080151

**Published:** 2023-07-28

**Authors:** Ozlem Cankaya, Natalia Rohatyn-Martin, Jamie Leach, Keirsten Taylor, Okan Bulut

**Affiliations:** 1 Department of Human Services and Early Learning, MacEwan University, Edmonton, AB T5J 4S2, Canada; rohatynmartinn@macewan.ca; 2 Department of Child and Youth Study, Mount Saint Vincent University, Halifax, NS B3M 2J6, Canada; jamie.leach@msvu.ca; 3 Department of Psychology, MacEwan University, Edmonton, AB T5J 4S2, Canada; taylork94@mymacewan.ca; 4 Centre for Research in Applied Measurement and Evaluation, University of Alberta, Edmonton, AB T6G 2R3, Canada; bulut@ualberta.ca

**Keywords:** play, cognitive development, toys, play objects, loose parts, loose parts play

## Abstract

Play is an integrative process, and the skills acquired in it—overcoming impulses, behavior control, exploration and discovery, problem-solving, reasoning, drawing conclusions, and attention to processes and outcomes are foundational cognitive structures that drive learning and motivation. Loose parts play is a prominent form of play that many scholars and educators explicitly endorse for cognitive development (e.g., divergent thinking, problem-solving). It is unique among play types because children can combine different play types and natural or manufactured materials in one occurrence. While educators and policymakers promote the benefits of loose parts play, no previous research has explored the direct relationship between preschool-age children’s indoor loose parts play experiences and cognitive development. We address this gap by bringing together the relevant literature and synthesizing the empirical studies on common play types with loose parts, namely object and exploratory, symbolic and pretend, and constructive play. We also focus on studies that examine children’s experiences through loose parts, highlighting the impact of different play types on learning through the reinforcement of cognitive skills, such as executive function, cognitive self-regulation, reasoning, and problem-solving. By examining the existing literature and synthesizing empirical evidence, we aim to deepen our understanding of the relationship between children’s play with loose parts and its impact on cognitive development. Ultimately, pointing out the gaps in the literature that would add to the body of knowledge surrounding the benefits of play for cognitive development and inform educators, policymakers, and researchers about the significance of incorporating loose parts play into early childhood education.

## 1. Introduction

Play is a foundational and universal phenomenon in the development of young children, often defined as an activity pursued for its own sake and mainly characterized by processes rather than end goals (Smith 2005). Following Burghardt (2005, 2010) and Pellegrini (2009), we define play as a process involving a range of intrinsically motivating activities for enjoyment. Although the exact definition of play is debated (Smith 2005; Wallerstedt and Pramling 2012; Whitebread et al. 2012; Zosh et al. 2018), there is consensus on children’s motivation for involvement in play for exploration and discovery and its exceptional complexity in inducing learning (Pyle et al. 2017; Smith 2005, 2010; Whitebread et al. 2012). Play is also an integrating process (Wood and Bennett 1997), where children draw upon and connect previous experiences, represent their ideas in different ways, imagine possibilities, explore, and create new meanings (Dockett and Perry 2007).

Researchers have explored specific types of play (e.g., pretend, construction, sensorimotor) and their capacity to enhance children’s cognitive development (Lillard et al. 2013; Wolfgang et al. 2001; Smith 2017). The more complex the play, the more it impacts development (e.g., pretend play; Beckwith et al. 1994; Lifter et al. 2011; Lillard et al. 2013; Zosh et al. 2022). What is evident is that children acquire foundational cognitive skills that drive learning during play, such as overcoming impulses through cognitive self-regulation, behavior control through emotional self-control, exploration and discovery, problem-solving, receptive and expressive language, social interaction, and attention to processes and outcomes (Park 2019; Wolfgang et al. 2001). Many researchers recognize play as a medium for learning and the foundation for exploration (Bergen 2009; Pramling Samuelsson and Johansson 2009; White 2012; Whitebread et al. 2017). There is growing global interest in loose parts play (LPP) to enrich children’s indoor experiences to motivate experimentation and learning (e.g., Beaudin 2021; Beloglovsky and Daly 2015, 2016; Caldwell 2016; Casey and Robertson 2016; Daly and Beloglovsky 2014; Eren-Öcal 2021; Gençer and Avci 2017; Rawstrone 2020; Sear 2016).

This literature review first provides an overview of loose parts play and highlights its unique characteristics. Subsequently, drawing upon existing research on play and cognitive development, we examine the impact of specific types of play with loose parts on the cognitive development of young children. Through a comprehensive synthesis of the available literature, we explore how different play opportunities influence cognitive capacities, including executive function, cognitive self-regulation, reasoning, and problem-solving. Our review underscores the crucial role of play in facilitating the development of fundamental cognitive abilities, which in turn have long-term implications for learning and cognitive outcomes. This literature review critically integrates research findings to shed light on the potential contribution of loose parts play to cognitive development, emphasizing the significance of play as a means of fostering cognitive skills through the utilization of these specific materials.

### 1.1. What Is Loose Parts Play?

LPP is defined as children’s play with open-ended and interactive materials (e.g., cardboard, shells, tires, sand, pompoms) not initially intended for play that can be manipulated limitlessly (Gull et al. 2019). LPP is an engaging form of play for children that offers complexity because children can combine different play types and various materials in one occurrence (Beaudin 2021). This form of play emphasizes materials that allow children to play in multiple ways and levels of complexity while experimenting, discovering, inventing, and having fun (Casey and Robertson 2016; Sear 2016). Indeed, LPP has many elements of free or unstructured play, as described by other researchers. These play types, like LPP, are often described as springboards for all subsequent learning, where children’s ideas, interests, and desires are respected, nurtured, and expanded into an ongoing, orderly, and recognizable curriculum incorporating knowledge from all disciplines (Van Camp 1972; UNICEF 2023). However, free play may include any unstructured activity that inspires a child to use their imagination without constant adult direction. Some examples of free play include children playing together in the backyard, where various activities, such as running, jogging, climbing, jumping, and fine motor movement, help the child develop speed, strength, stamina, flexibility, and coordinative abilities. Likewise, unstructured play may resemble LPP, allowing children to explore, create, and discover without predetermined rules or guidelines. Like free play, however, this is open to a broad scope of activities, including artistic or musical games, imaginative games (e.g., making a fort with boxes or blankets), dressing up or playing make-believe, or exploring new spaces like woods, backyards, parks, and playgrounds.

### 1.2. What Is Unique about Loose Parts Play?

Nicholson (1972) coined the term loose parts and described the importance of interactive materials that can have many affordances.. According to Affordance Theory (Gibson 1979), the world is perceived as an object of possibilities for action or affordances. In terms of materials, affordances refer to how an object or material can be used or interacted with. Children’s LPP can involve a variety of materials: everyday synthetic or natural materials, reusable and upcycled materials, and commercial toys that may promote thinking in Science, Technology, Engineering, Arts, and Mathematics (STEAM) (Beloglovsky and Daly 2016; Drew and Rankin 2004; Bairaktarova et al. 2011). Play materials with many affordances provide children with more opportunities to learn and develop new skills through their play.

We know that children’s play frequently involves objects, materials, or toys (Gull et al. 2020; Tizard et al. 1976), and play themes generally follow the ideas inherent in the materials and toys available (Pellegrini and Smith 1998; Pellegrini and Perlmutter 1989; Smith and Connolly 1980). Thus, LPP is unique because the materials are clearly defined by their affordances compared to those used in other play types (e.g., musical, pretend). It is important for children’s play materials to have many affordances because it allows for a wide range of exploration and creativity. For example, a simple wooden block can be used as a building material, a tool for stacking and balancing, or a prop in imaginative play. Each of these uses can offer a different learning experience and help children develop a range of skills, such as problem-solving, spatial reasoning, and fine motor skills.

According to Trawick-Smith et al. (2014), quality play encourages children to be involved in critical learning and cognitive development elements such as self-regulation, make-believe, problem-solving, and creative expression. High-quality play offers many educational benefits such as problem solving and learning (Bergen 2006; Gronlund 2010). When children have access to play materials with many affordances, they are more likely to engage in open-ended and imaginative play that reinforces these educational benefits. LPP allows children to explore their interests and ideas to develop their creativity and self-expression. Additionally, having access to a variety of play materials with many affordances can help with attention shifts and increase engagement in play, which is essential for children’s development and well-being. Consequently, materials used in LPP are more likely to fulfill quality play opportunities.

Researchers have shown that materials with many affordances in children’s play, such as those used in LPP, also have developmental benefits (Guyton 2011; Bairaktarova et al. 2011; Kiewra and Veselack 2016; Segatti et al. 2003; Shabazian and Soga 2014). For example, these materials can inspire, maintain, and spark ideas, support children using symbolic skills to transform ideas into scenarios during play, draw social interaction into a shared play sphere, promote self-esteem, emotional well-being, and resilience, and foster children’s higher mental processes, such as thinking or internal dialogues (Pellegrini and Bjorklund 2004; Pepler and Ross 1981; Mundy and Newell 2007; Drew and Rankin 2004; Whitebread et al. 2012; Schaefer 2016). Not all toys and materials are equally effective in promoting engaging play, especially for children of different ages (Cutter-Mackenzie and Edwards 2013; Trawick-Smith et al. 2011). While it is difficult to define engaging play by age, one parameter can be helpful to define play that sustains children’s attention over a period of time with elaborate themes and ideas. Open-ended materials and toys that do not suggest a play theme allow for many kinds of play, including constructive and pretend play, that can lead to positive outcomes (Trawick-Smith et al. 2015). Trawick-Smith et al. (2015) also found that play materials and toys with many affordances do not serve younger children well but promote engaging play for older ones. Younger children perform their most frequent pretend-to-play with realistic toys. Furthermore, everyday objects and natural materials can foster cause-and-effect or trial-and-error explorations and positively influence children’s cognitive development by sparking imagination, creativity, and motivation for further exploration and learning (Bairaktarova and Evangelou 2012; Kiewra and Veselack 2016).

In addition, Howe et al. (2022) investigated how open-ended versus closed-ended toys impact children’s pretend play. They found that open-ended toys are particularly important in supporting children’s play and learning, as they encourage divergent and convergent thinking, imagination, and problem-solving skills. The nature of toys determines children’s patterns of communication and behavior. The authors concluded that although the toy themes are somewhat suggestive, they may not promote similar behaviors and outcomes in pretend play. Therefore, the type of toys and play materials children can access can significantly impact their play experiences, the play types they involve in, and their learning outcomes. Open-ended materials and toys that allow for many kinds of play have been found to have developmental benefits, foster creativity, and encourage problem-solving skills. Thus, considering play materials with many affordances provide opportunities for LPP to promote children’s growth and development. Researchers have heavily investigated the developmental benefits of playing with individual open-ended materials, i.e., play with blocks, LEGO^®^, or sand in isolation (e.g., Kiewra and Veselack 2016; Schulz and Bonawitz 2007; Segatti et al. 2003; Shabazian and Soga 2014; Zippert et al. 2019). However, LPP can involve interactive materials used simultaneously in various play types (Casey and Robertson 2016; Daly and Beloglovsky 2014).

### 1.3. What Is the Status of Research on Loose Parts Play?

As already highlighted, broad interest in LPP to enrich children’s play experiences has grown (Beaudin 2021; Beloglovsky and Daly 2015), with claims to be a developmental foundation for creativity, problem-solving, and divergent thinking. However, research has not kept pace with the enthusiasm of childcare professionals and policymakers (e.g., Nova Scotia Department of Education and Early Childhood Development 2018). Recent systematic and scoping reviews document that, thus far, there are only a handful of empirical studies on children’s LPP, and the focus on the developmental benefits of this type of play is limited, especially for cognitive development (Gibson et al. 2017; Gull et al. 2019; Houser et al. 2016). Instead, researchers have focused primarily on outdoor LPP, examining physical and social development (Houser et al. 2016; Maxwell et al. 2008; Spencer et al. 2019). Indoor play environments offer unique opportunities for young children to engage in imaginative, creative, and sensory-rich activities. However, despite the prevalence of indoor play spaces and the common recommendation of loose parts play for the preschool age group, there is limited scientific research available to support these practices (Gibson et al. 2017). Studies on young children’s indoor LPP are limited to a few non-empirical studies (Beaudin 2021; Sear 2016; Rawstrone 2020). No empirical work considers children’s cognitive functioning (e.g., verbal IQ, executive function). Furthermore, the influence of critical factors such as a child’s age, family income, and educational attainment on parent/child play types, duration, and engagement with loose parts has not yet entered the research dialogue. Thus, there is a noticeable research gap when it comes to understanding the specific benefits of indoor play for children under the age of 6.

The types of play children commonly utilize with loose parts have yet to be documented or explained. Such knowledge would support understanding which materials are most conducive to specific types of progressively complex play, allow children to design their own learning goals, and prepare young children for learning. The evidence that illustrates the developmental benefits of this type of play is very limited. For example, Gibson et al. (2017) robustly indicated the lack of research on the benefits of LPP. They highlighted that little is known about how LPP influences children’s development beyond physical and social domains (Flannigan and Dietze 2017; Gibson et al. 2017). Early studies have focused narrowly on children’s outdoor LPP (Flannigan and Dietze 2017; Gull et al. 2019; Houser et al. 2016; Spencer et al. 2019; Olsen and Smith 2017) and mostly on physical and social development (Dobbins et al. 2013; Engelen et al. 2013; Flannigan and Dietze 2017; Houser et al. 2016; Maxwell et al. 2008; Ridgers et al. 2011; Spencer et al. 2019). Furthermore, the existing empirical studies focus mainly on older children (c.f. McLoyd 1983; Maxwell et al. 2008; Oncu et al. 2015). Empirical work on young children’s indoor LPP types and their relationship to preschool-age children’s cognitive skills in the current literature is crucially lacking.

**Key Points**

1. Loose Parts Play (LPP) focuses on utilizing materials that offer multiple possibilities, enabling children to engage in diverse play experiences and develop cognitive capacities like executive function and cognitive self-regulation. LPP provides flexibility and adaptability, allowing children to manipulate, combine, and transform loose parts in countless ways, thereby facilitating open-ended play experiences.
2. Research on LPP is limited, especially regarding its developmental benefits for cognitive development. Existing studies primarily focus on outdoor LPP and its impact on physical and social development. More research is needed, particularly on young children’s indoor LPP and its influence on cognitive functioning, considering factors such as the child’s age or family socio-economic status.
3. Play, including loose parts play, is fundamental in childhood and significantly impacts children’s cognitive development. The open-ended nature of loose parts play fosters divergent thinking and flexible problem-solving approaches.
4. Symbolic and pretend play promote cognitive skills such as symbolic substitution, dual representation, language development, executive function, self-regulation, and problem-solving. Loose parts play also supports constructive play, stimulating cognitive advancement, problem-solving, and higher-level thinking, while enhancing social interaction and communication skills.
5. Further research is needed to explore the specific impact of loose parts on children’s play while considering the effects of a child’s cognitive development, age, socio-economic status, and cultural differences.

## 2. How Do Specific Types of Play with Loose Parts Impact Young Children’s Cognitive Development?

### 2.1. Object and Exploratory Play with Loose Parts

Object play is an infant or child’s playful exploration of an object and or engagement with it to learn about its properties (Hughes 2021; Smith 2010) and progresses from early sensorimotor explorations to symbolic objects (i.e., using objects to represent other objects, such as a banana as a telephone) for communication, language, and abstract thought (Yogman et al. 2018). Hughes (2021) describes object play as encompassing problem-solving, considering it ‘problem-solving play’. Problem-solving can be defined as a cognitive process through which individuals identify, analyze, and apply solutions to overcome obstacles or challenges that hinder the achievement of a desired goal. It involves systematically exploring and evaluating different strategies, information, and resources to find the most effective and efficient way to solve a problem or address a complex situation. Certainly, object play is viewed as a window to cognitive processes—a means for children to express their knowledge and interpret new knowledge by exploring objects at hand (Lifter et al. 2022). Piaget and Cook (1952) described children’s object play as originating from sensorimotor explorations and termed it sensorimotor play. This early exploratory play is the first form of object play and typically begins around five months of age (White 2012). Within a year, simple reflexes turn into intentional, coordinated movements of exploration. Concerning cognitive developmental stages, Piaget and Cook (1952) postulated that children’s external actions to understand how the world operates eventually become internal representations.

Through manipulating objects, toddlers begin to have cognitive representations of the world that can be evident in how they relate to the representations of objects and people around them. For instance, if a young child wants an obstructed object, they reach for it directly. These trial-and-error sessions for younger children transform into deliberate planning for older children (Smith 2010). Pellegrini (2013) describes the benefits of children’s object play as part of learning behavioral “modules”. He emphasized that modules are novel and recombined behavior and cognitive routines constructed by individuals in response to new ecological demands. With experience, these diverse behavioral routines become more focused and relevant to the environment. Pellegrini (2013) speculated that children’s play experiences with objects generate behavioral modules.

Object play has a strong presence in children’s lives and remains a large part of the daily routine, occupying approximately 10–15% of children’s waking hours by conservative estimation (Smith and Connolly 1980). Its cognitive developmental contribution includes learning about the nature of objects, problem-solving, creativity, and foundational skills for science, technology, engineering, and mathematics (White 2012). Through exploratory object play, children are introduced to the function of objects and how to control them (Bjorklund and Gardiner 2011). They use object exploration to test their hypotheses about their environments and how those objects operate by touching and manipulating parts of the toy (Schulz and Bonawitz 2007), even in infants (Baldwin and Moses 1994; Gweon 2012; Schulz 2015). Indeed, the quality of the evidence children observe affects their exploratory play. Schulz and Bonawitz (2007) found that preschool children distinguish confounded and unconfounded evidence and selectively engage in more exploration when the causal structure of events is ambiguous. Thus, the exploratory play of even very young children appears to reflect some of the logic of scientific inquiry and give them a basis to practice the life-long skill of learning about the properties of and uses for objects that they can touch, hear, and see. More crucially, it helps them to make inferences about properties that are not as easy to ascertain (Gweon 2012; White 2012).

Given play’s imaginative and flexible nature, another core cognitive skill facilitated by exploratory and object play is problem-solving (Cankaya 2022), particularly divergent problem-solving skills. By engaging in problem-solving tasks, individuals develop their analytical capabilities, enhance their adaptability, and acquire valuable problem-solving skills that can be applied in various aspects of life and learning (Lillard et al. 2013). For example, Pepler and Ross (1981) assigned young children to play with a puzzle, considered a convergent toy due to its single solution to the problem. In another condition, children were offered a multiple-option block set, considered a divergent toy. In later tasks, children who played with the blocks were more innovative and flexible in their problem-solving approaches than their peers who played with convergent toys. The researchers emphasized that children benefit from divergent experiences and that those experiences can be transferred and generalized more broadly. They also found that children who played with the divergent toys were generally successful on various divergent and convergent problem-solving tasks, suggesting that engaging in divergent playful activities might instill the idea of numerous creative solutions to a problem (Pepler and Ross 1981; White 2012).

Furthermore, Solis et al. (2017) documented that experiencing and manipulating physical principles through objects allows young children to formulate scientific intuitions, serving as potential precursors to learning in STEAM subjects. Crucially, this supports children’s reasoning skills with materials. Through naturalistic observations of preschool children’s free play, they demonstrated that children encountered various physics concepts while engaging in spatial–mathematical activities. This occurred as children engaged in planning and executing play sequences, solving problems, and exploring the objects available. During play, children discover physical principles through object affordances (Nicholson 1972). Similarly, Bjorklund and Gardiner (2011) asserted that children could explore and learn about the properties of and uses for objects they can see, touch, and hear through solitary object play.

Object play has been found to have a positive impact on children’s visuospatial skills, which are crucial for their numerical reasoning abilities (Caviola et al. 2014; Fanari et al. 2019; Holmes et al. 2008; LeFevre et al. 2010; Sella et al. 2016). Several studies have directly linked early object play to better math outcomes (Caldera et al. 1999; Verdine et al. 2019; Wolfgang et al. 2001). Longitudinal research conducted by Wolfgang et al. (2001) suggests that engaging in complex object play during early childhood can lay the foundation for later mathematical understanding in formal learning contexts. Additionally, Verdine et al. (2019) explain that tangible toys with various geometric shapes can enhance children’s spatial language use and facilitate interactions between adults and children, thereby supporting the development of early geometric knowledge. These findings highlight the significance of object play in promoting children’s mathematical skills and the importance of providing them with opportunities to engage with toys and materials that foster spatial thinking and geometric understanding.

Riede et al. (2021) created a framework for understanding the role of play objects and object play for innovative behavior. They emphasized that children’s play strongly reflects adult behaviors, and play that involves imagination encourages children to explore the consequences of potential benefits of social and technological action schemata before enacting them. Some toys offer a powerful opportunity as an innovation primer, allowing children to explore the complex, emergent mechanical and material affordances of associated adult technologies (Lancy 2017). Riede et al. (2021) suggested that play objects offered by adults to their youngsters significantly affect children, adolescents, and young adults’ possibility of becoming innovative. Teachings and pedagogical interventions may help maintain long-term traditions, and playing with objects may function as a primer for innovation (Riede et al. 2021). Even children’s brief frequencies of experimentation increase propensities to innovate in late childhood, adolescence, and later life. These trial-and-error activities scaffold children to develop creativity and strategies to tackle novel problems successfully.

Children involve loose parts in their object and exploratory play by incorporating and manipulating objects with multiple uses (Scott-McKie and Casey 2017). Loose parts can be moved, combined, designed, redesigned, taken apart, and put together in endless ways (Nicholson 1972). Children involve loose parts in their object play differently from other objects due to the unique characteristics and possibilities they offer (Nicholson 1972; Beloglovsky and Daly 2015). Loose parts include various materials in combination (Cankaya 2023) that are predominantly open-ended and can be used in multiple ways, while other objects may have a specific intended purpose or limited functionality. In that regard, loose parts offer greater flexibility and adaptability in play. They can be combined, arranged, and modified in various ways, allowing children to create unique structures, designs, or scenarios.

In contrast, other objects may have limited possibilities for manipulation or customization. For example, a toy car typically serves a specific role and function. In contrast, a loose part, such as a stacking cup, can be a building element, a prop in pretend play, or a part of a sorting activity. Children can explore and engage in both object and exploratory play, but loose parts give them more options for how objects can be used in various ways. Also, children can transform and repurpose loose parts based on their imagination, whereas other objects may have predetermined uses or fixed representations. Thus, loose parts give children more opportunities for flexibility and adaptability in object and exploratory play, as they need to figure out how to use loose parts effectively, experiment with different combinations, and overcome challenges. Other objects often have a more prescribed use (e.g., puzzles; Scott-McKie and Casey 2017), limiting the need for problem-solving in the same way. Loose parts give children greater freedom, flexibility, and open-ended possibilities compared to other objects, making them unique components of children’s play experiences.

### 2.2. Symbolic and Pretend Play with Loose Parts

Symbolic and pretend play activities are characterized by an ‘as-if’ stance (Garvey 1990), and the playful set of behaviors and activities often involve nonliteral actions (Weisberg 2015). In the context of pretend play, the child taking on the role of the pretender consciously and purposefully projects a mentally represented alternative onto the current situation, fostering a playful atmosphere (Lillard et al. 2013). Children’s involvement in symbolic or pretend play is depicted by an active transformation of the here and now. It involves a living agent who is aware that they are pretending, a reality that is pretended, and a mental representation projected onto reality. For example, a child may use a stick as a sword and pretend to strike a playmate who then pretends to be injured. This type of play is alternatively called imaginary play or pretense. Furthermore, Holmes et al. (2019) definition of creative play encompasses pretend and symbolic play, wherein children think creatively through these types of play; in a sense, creative play may occur as a result of pretend or symbolic play.

Like other kinds of play, pretend play is connected to children’s cognitive development although, uniquely, it appears to be distinctively human (Smith 2010; Whitebread et al. 2017). Symbolic substitution is a cognitive benchmark for young children that manifests as language skills and emerges after the first 12 months (Whitebread et al. 2017). Around this time, children start mastering various symbolic systems such as spoken language, numbers, and music. Some examples are when a child pretends a cup is a party hat (not immediately clued in the environment), locating objects from a map, reading words, and understanding their reference points. This dual representation is the ability to think about an object in different ways simultaneously (DeLoache 2000; Uttal et al. 2009). This ability first appears around age 2, increases rapidly, and signals an awareness that the child has begun representational activity (Whitebread et al. 2017). Therefore, pretending and language development grow with children’s ability to think symbolically. Children’s symbolic or pretend play gradually includes more complex schemes in the appearance of sociodramatic or role play. Consequently, preschoolers are observed to spend more time in pretend play than younger children (Howes and Matheson 1992).

Researchers have also demonstrated that children’s pretend play has both short-term and long-term benefits for cognitive development (Copple and Bredekamp 2009, p. 15; Lillard et al. 2013; Savina 2014). In particular, a significant body of evidence shows that children involved in complex pretend play have executive function skills (Coelho et al. 2020; Garon et al. 2008; Germeroth et al. 2019; Kelly et al. 2011; Walker et al. 2020). Executive function (EF) is a set of high-level cognitive processes that facilitate new ways of behaving and optimize one’s approach to unfamiliar circumstances (Baddeley 2002; Barkley 2001; Cumming et al. 2022; Diamond 2013; Garon et al. 2008; Happaney and Zelazo 2022; Zelazo et al. 2003). It includes basic cognitive processes such as attentional control, cognitive inhibition, inhibitory control, working memory, and cognitive flexibility. EF is often explained with the analogy of an “air traffic control system at a busy airport that safely manages the arrivals and departures of many aircraft on multiple runways” (Center on the Developing Child at Harvard University 2011). These mental processes play a critical role in a person’s ability to manage daily life tasks such as sustaining attention, keeping goals and information in mind, refraining from responding immediately, resisting distraction, tolerating frustration, considering the consequence of different behaviors, reflecting on past experiences, planning for the future, and balancing multiple tasks successfully (Diamond 2013; Garon et al. 2008; Zelazo et al. 2016). Thus, they are necessary for the cognitive control of behavior: selecting and successfully monitoring behaviors that facilitate the attainment of chosen goals.

Although the direction of the relationship between children’s EF and pretend play is still subject to debate (Lillard et al. 2013), several play-centered interventions have successfully enhanced children’s executive function (Coelho et al. 2020; Elias and Berk 2002; Thibodeau et al. 2016; Walker et al. 2020). Children involved in short amounts of pretend play regularly (e.g., less than 10 min daily) show improvements in performance on subsequent executive function tasks (Carlson and White 2013). Walker et al. (2020) developed a pre–post design intervention study where educators embedded targeted activities and role-playing with a problem to solve collectively over ten weeks, resulting in significant behavior improvements in children’s executive function performance. Similarly, Kelly and colleagues observed 4- and 5-year-old children in their free play to explore the role of inhibitory control in symbolic play. They found that greater inhibitory control positively correlated with more symbolic play. These studies indicate that encouraging children to engage in pretend play, particularly more mature forms, could be a natural vehicle by which adults can promote EF and self-regulation (Lillard et al. 2013; White 2012).

Furthermore, growing evidence shows the development of self-regulation skills through pretend play (Elias and Berk 2002; Savina 2014; Slot et al. 2017). Self-regulation, the ability to understand and manage one’s behavior and reactions (Savina 2014), develops rapidly in the first years of life and continues to develop into adulthood. EFs are presumed to be the general forms or classes of self-directed actions we use in self-regulation (Barkley 2001). In children’s play, self-regulation may manifest in a variety of ways, such as regulating reactions to intense emotions such as frustration or excitement, calming down after something exciting or upsetting happens, focusing on a task, refocusing attention on a new task, controlling impulses, or learning a range of behaviors to engage in social play (i.e., play with others; Eisenberg and Sulik 2012; Post et al. 2006). EF and self-regulation may produce an overall net maximization of social consequences when considering response alternatives’ immediate and delayed outcomes. They are also instrumental in purposive, intentional behavior such as learning, experimenting with new ideas, or verbal communication (Barkley 2001). Significantly, Weintraub et al. (2013) showed that EF skills build throughout childhood and adolescence, with early childhood having the most dramatic growth. Since proficiency in numerous EFs decline later in life (Weintraub et al. 2013), mastering such skills during the early years may be essential for daily life functioning in later adulthood.

Slot et al. (2017) differentiated between cognitive and emotional self-regulation and investigated how 3-year-olds demonstrated each in a naturalistic play setting. ‘Hot’ executive functions refer to self-management skills when emotions run high, while ‘cool’ executive functions refer to skills when emotions are not a factor. They found that in neutral or cool situations, children’s EF typically includes working memory, inhibitory control, and cognitive flexibility and that 3-year-olds showed aspects of cognitive and emotional self-regulation (Blair and Ursache 2011; Slot et al. 2017). Cool EFs appeared significantly related to emotional self-regulation, whereas hot EFs were not significantly related to cognitive or emotional self-regulation. Specific to our discussion in this paper, they found that the quality of pretend play was strongly associated with cognitive self-regulation and, to a lesser extent, emotional self-regulation. These findings further suggest that pretending may encourage the flexible thinking required for children to overcome impulses and successfully control cognitive behaviors (Slot et al. 2017; White and Carlson 2016).

Loose parts allow children to use their imagination and assign symbolic representations to objects (e.g., please compare building a house with Magna-Tiles versus including a dollhouse in children’s pretend play; Gronlund 2010). They can transform a simple loose part, such as a stick or a fabric, into a pretend object with multiple meanings. For example, a stick can become a magic wand, a fishing rod, or a sword. Other objects often have specific and fixed representations that limit their transformative potential (e.g., Elsa’s or Harry Potter’s magic wand). Children can adapt and incorporate loose parts into various roles and scenarios during pretend play (Gronlund 2010; Scott-McKie and Casey 2017). They can easily change the purpose and function of loose parts based on the evolving storyline or their imaginative needs. In contrast, other toys and play materials often have predefined roles and are less adaptable to different pretend play situations. Halfway through a pretend or symbolic play scenario, if children see the idea or opportunity, they can combine loose parts, rearrange them, or add additional elements to create props or set designs for their imaginative scenarios.

Furthermore, loose parts can facilitate the use of language, in particular, productive language through narrative expansion in pretend play. Children can introduce new loose parts into their play to enrich the storyline or add complexity to their pretend world. They can bring in additional loose parts to represent characters, objects, or settings, enhancing the depth and breadth of their imaginative play. An active play partner in pretend or symbolic play can benefit young children’s LPP, resulting in longer and more complex play episodes than when they play alone (Balfanz et al. 2003; Ramani and Eason 2015; Schmitt et al. 2018). Children frequently involve others in their play in early learning environments. Pramling Samuelsson and Johansson (2009) explored why children involve teachers in their play and learning through video recording children’s play in preschool and primary schools. They found five reasons for involving teachers: getting help from the teacher, acknowledging teachers as competent persons, making the teachers aware of other children breaking the rules, getting information about and confirmation of how things work, and involving teachers in play. Pramling and colleagues reasoned that children see educators as knowledgeable and that they can contribute to their learning processes. As children age, they mobilize educators as resources to learn about something or ask questions to expand and continue with the task. Children’s time playing with others can be a time to learn new skills, practice existing abilities, and build interests (Ramani and Eason 2015). The continuum of playful learning shows the different levels of social interaction involved in experiences. The flexible component of loose parts allows children to play, explore, and discover independently in their pretend play. By involving loose parts in their pretend play, children have the freedom to transform objects, adapt them to different roles, and engage in imaginative and flexible play scenarios. The open-ended nature of loose parts can promote creativity, symbolic representation, narrative expansion, and collaborative play experiences that may differ from the limitations imposed by other objects.

### 2.3. Constructive Play with Loose Parts

Constructive play, characterized by manipulating materials to create things, is a commonly observed form of play during preschool and kindergarten free play periods (Gronlund 2010; Park 2019). It typically begins around the age of 2 and becomes the most prevalent form of play between the ages of 4 and 6, accounting for a significant portion of children’s playtime (Rubin 2001). During constructive play, children experiment with materials, manipulating and constructing objects (Maxwell et al. 2008). They create something by combining basic elements, arranging them in various ways, and achieving a goal through these processes (Forman 2006, 2021). Examples of constructive play include building forts, stacking blocks, constructing LEGO sets, making sandcastles, and shaping playdough figures, which can be both static and dynamic in nature. Constructive play with basic elements, such as blocks, offers opportunities for children to develop cognitive skills, including symbolic awareness and problem-solving abilities (Han and Park 2010). It allows children to produce patterns, objects, and functional systems and engage in pretend sequences, fostering creativity and imaginative thinking (Forman 2006). Moreover, constructive play provides insights into children’s thinking processes as they pretend, invent, improvise, and design their own rules at their own pace (Forman 2006). Overall, constructive play stimulates cognitive advancement and promotes higher-level thinking in children (Ness and Farenga 2016; Park et al. 2008).

Children’s play themes generally follow the ideas inherent in the materials and toys available. However, materials and toys used for children’s play have changed significantly over the years, reflecting societal changes, technological advancements, and shifts in understanding child development (Cankaya 2023). Loose parts can be offered in many combinations, but the impact of material choice on children’s play types and engagement is unknown (Gibson et al. 2017). Children can incorporate loose parts materials into their constructions, adding a sense of authenticity and connection to the natural world (Beloglovsky and Daly 2016). They also need to consider the spatial relationships, balance, and structural integrity of their constructions (Scott-McKie and Casey 2017).

According to researchers, the most prominent cognitive skills involved in early childhood science involvement in constructive play are problem-solving, critical thinking, and scientific inquiry using trial and error (Campbell et al. 2018; Soylu 2016; Yücelyiğit and Toker 2021). This can increase children’s understanding of geometry, physics, and architectural principles. Other construction toys (e.g., Lincoln Logs) may not incorporate other toys in the same way that would allow various design elements that originally did not have a place in the construction. Ness and Farenga (2016) suggest that the specific qualities of some play materials (e.g., blocks, bricks, and planks) may help establish the scientific, mathematical, and technological foundations for children’s cognitive development, as opposed to scripted play properties may have the opposite effect that the use of products manufactured with specialized, commercialized themes prevents children from self-regulation and even ideation. Loose parts often encourage collaboration and social interaction, particularly with peers during constructive play.

Collaborative building requires them to create and establish joint goals using receptive and expressive language, such as what they would build and how they would build it (Vriens-van Hoogdalem et al. 2016). It also necessitates communicating actions, representations of the blocks, and the significance of the structures they create. During peer play, children must also coordinate their behaviors, communicate effectively to establish the interaction’s goals and rules, and work through disagreements by understanding each other’s views (Pellegrini 2009; Ramani et al. 2014). Through discussion, children attempt to resolve their differing perspectives and advance their understanding of difficult, complex problems. Thus, peer involvement and cooperative play activities are characterized by this common understanding of the goals and processes to execute them (Bratman 1992). Children can share loose parts, negotiate roles, and work together to create and develop their pretend play scenarios. Other objects may not foster the same level of collaboration and shared imagination. Children engage in open-ended exploration, creative problem-solving, and collaborative building experiences that differ from other objects or toys’ more structured and limited possibilities. Thus, loose parts’ constructive play capacity is qualitatively different from other play materials and toys. Table 1 below presents research findings that establish connections between specific play types, as explained in this article, and their impact on young children’s cognitive development. Each play type contributes to various aspects of cognitive growth, highlighting the diverse benefits associated with different types of play.

## 3. Conclusions and Future Directions

Play is fundamental in childhood and consumes a large portion of children’s unstructured time (Haight and Miller 1993). There is a clear developmental progression of children’s ability to engage in play from sensorimotor play during infancy to the emergence of pretend play in early childhood. A child’s engagement in various types of play with open-ended materials and play partners leads to qualitative changes in their play complexity and core cognitive skills that are critical in learning and motivation, even in adulthood.

Children’s cognitive development proceeds rapidly in the early years, which mirrors the dynamic changes in their play. The ability to play, specifically pretend play, appears to be an early expression of children’s understanding of symbols and symbolic relations (Piaget 1962). The discussion regarding the direction of the relationship between play and cognitive development is complex (e.g., Duncan and Tarulli 2003). However, there is growing evidence that some play types have the potential to facilitate core cognitive functions (Coelho et al. 2020; Garon et al. 2008; Germeroth et al. 2019; Kelly et al. 2011; Walker et al. 2020). In particular, play experiences facilitate the development of various cognitive skills, including executive function, problem-solving, and critical thinking (Andersen et al. 2022; Hirsh-Pasek et al. 2004, 2009, 2020; Jaarsveld et al. 2012; Koerber et al. 2005; Lillard et al. 2013; Schulz and Bonawitz 2007; Weisberg et al. 2016; Whitebread et al. 2017). As children acquire these complex cognitive capacities, their play becomes more multifaceted, thus reflecting and supporting the underlying mechanisms for core cognitive skills.

Play serves as a foundation for the development of lifelong learning skills. Individuals with well-developed executive function skills are better equipped to engage in independent learning, self-directed study, and effective time management. These skills also support academic achievement by facilitating sustained attention, impulse control, and emotional regulation during challenging tasks. Moreover, researchers also document that play facilitates cognitive self-regulation skills (Elias and Berk 2002; Savina 2014; Slot et al. 2017), which are crucial skills for learning, school achievement, and building and maintaining social relationships. Executive function and cognitive self-regulation are necessary for optimal cognitive development, as they provide the cognitive abilities needed to navigate complex tasks, set goals, adapt strategies, and make informed decisions. Strengthening these skills during early development lays a solid foundation for continued learning and success throughout life. While further research is needed to deepen our understanding, the existing evidence highlights the potential of engaging in specific types of play with indoor loose parts to positively influence young children’s cognitive development and equip them with essential skills for lifelong learning.

Play provides opportunities for children to actively engage in activities that require them to plan, strategize, and make decisions, thereby enhancing their cognitive abilities. Furthermore, play serves as a catalyst for learning, allowing children to explore their interests, acquire new knowledge, and develop a deeper understanding of concepts. The intrinsic motivation inherent in play promotes active engagement, curiosity, and a positive attitude toward learning, in particular, if the materials are conducive to various types of play (Trawick-Smith et al. 2015). Although research on loose parts play is currently limited, studies focusing on specific types of play with open-ended materials provide compelling evidence that engaging in play with indoor loose parts can potentially have a significant impact on the cognitive development of young children (White 2012; Baldwin and Moses 1994; Bjorklund and Gardiner 2011; Caviola et al. 2014; Fanari et al. 2019; Gweon 2012; Holmes et al. 2008; LeFevre et al. 2010; Schulz and Bonawitz 2007; Schulz 2015; Sella et al. 2016).

Researchers have consistently shown that play fosters cognitive skills such as problem-solving and reasoning. The open-ended nature of loose parts play promotes divergent thinking and flexibility in problem-solving approaches. Children manipulate and interact with a variety of loose parts, allowing them to experiment, explore, and make connections, which supports their cognitive development. Moreover, through this type of play, children develop cognitive flexibility, innovation, and a deeper understanding of cause-and-effect relationships. The versatility and adaptability of loose parts play enable children to engage in self-directed learning experiences, promoting agency in their cognitive growth. Educators and parents should recognize the value of incorporating indoor loose parts play into children’s environments as a means to enhance their cognitive development and promote holistic learning experiences.

Discussing children’s play in international and diverse communities requires careful consideration of social, cultural, and political contexts impacting children’s lives (Shimpi and Nicholson 2014; Thibodeau-Nielsen et al. 2020). Adults (e.g., caregivers, parents, educators) must provide children with balanced opportunities for different kinds of play (e.g., constructive, pretend play) to nurture overall development. Providing a variety of materials to stimulate different types of play is necessary. For example, offering a variety of loose parts in rotating order could support cognitive development through quality play and engagement opportunities. It is also essential for adults to play with children on children’s terms. While there are benefits for children to be left on their own to free play, there are positive benefits of some active adult involvement in the play, such as supporting longer, more complex episodes of play (Ward 1994). Lastly, ensuring equity in all children’s access to play and play materials is essential for future success as a society. Equal access to play is vital for children’s sense of belonging and ensures all children can fulfill their potential for lifelong learning and success.

As society continues to change, research on play must be mobilized to ensure early learning educators, families, community advocates, and health and education professionals draw on the most current evidence to optimize children’s development and support their learning and cognitive growth (Barnett and Owens 2015). In particular, given the popularity of LPP, it is vital to investigate further its specific impacts on children’s development, such as the relationship between children’s cognitive development and LPP duration and complexity and the possible unintended outcomes of LPP on children’s cognitive, social, and physical development.

Furthermore, relatively little is known about the effects of socio-economic status and ethnic and cultural differences in children’s play. Further research on culturally specific play materials, their impact on children’s growth, the role of adults in play across cultural contexts, and even types of play in multicultural communities would provide much-needed context and understanding for professionals and educators working across or in mixed cultural communities. These areas represent just a few that are critical for investigation. Knowing how to promote learning through play must recognize the whole play continuum to ensure optimal conditions for children’s growth and development.

## Figures and Tables

**Table 1:** Research connecting specific play types to young children’s cognitive development.

		Cognitive Development			
Play Type	Executive Function	Self-Regulation	Reasoning	Problem Solving	Overall Cognitive Development
Object and Exploratory			Riede et al. 2021; Schulz and Bonawitz 2007; Solis et al. 2017	Hughes 2021; Lifter et al. 2022; Solis et al. 2017; White 2012	Andersen et al. 2022; Baldwin and Moses 1994; Bjorklund and Gardiner 2011; Caldera et al. 1999; Caviola et al. 2014; Fanari et al. 2019; Gull et al. 2020; Han and Park 2010; Happaney and Zelazo 2022; Hirsh-Pasek et al. 2020, 2004; Koerber et al. 2005; Schaefer 2016; Schmitt et al. 2018; Sear 2016; Trawick-Smith et al. 2014; Ward 1994; White and Carlson 2016
Symbolic and Pretend	Blair and Ursache 2011; Carlson and White 2013; Coelho et al. 2020; Elias and Berk 2002; Savina 2014; Slot et al. 2017; Thibodeau et al. 2016; Walker et al. 2020; White and Carlson 2016	Blair and Ursache 2011; Carlson and White 2013; Coelho et al. 2020; Elias and Berk 2002; Savina 2014; Slot et al. 2017; Thibodeau et al. 2016; Walker et al. 2020; White and Carlson 2016	Coelho et al. 2020; Garon et al. 2008; Germeroth et al. 2019; Kelly et al. 2011; Walker et al. 2020	Andersen et al. 2022	Copple and Bredekamp 2009; Hirsh-Pasek et al. 2020, 2004, 2009; Lillard et al. 2013; Savina 2014; Smith 2010; Whitebread et al. 2017
Constructive				Campbell et al. 2018; Soylu 2016; Yucelyigit and Toker 2021	Balfanz et al. 2003; Caldera et al. 1999; Han and Park 2010; Ness and Farenga 2016; Park et al. 2008; Park 2019; Ramani et al. 2014; Schmitt et al. 2018; Verdine et al. 2019; Wolfgang et al. 2001

## Data Availability

Not applicable.
